# Classification of EEG Signals Using Neural Network for Predicting Consumer Choices

**DOI:** 10.1155/2022/5872401

**Published:** 2022-07-20

**Authors:** K. Sheela sobana Rani, S Pravinth Raja, M. Sinthuja, B Vidhya Banu, R. Sapna, Kenenisa Dekeba

**Affiliations:** ^1^Department of Electronics and Telecommunication Engineering, Karpagam College of Engineering, Coimbatore, India; ^2^Presidency University, Bangalore, India; ^3^M.S.Ramaiah Institute of Technology, Bangalore, India; ^4^KNS Institute of Technology, Bangalore, India; ^5^Department of Food Process Engineering, College of Engineering and Technology, Wolkite University, Wolkite, Ethiopia

## Abstract

EEG, or Electroencephalogram, is an instrument that examines the brain's functions while it is executing any activity. EEG signals to aid in the identification of brain processes and movements and are thus useful in the detection of neurobiological illnesses. Pulses have a very weak magnitude and are recorded from peak to peak, with pulse width ranging from 0.5 to 100 V, which is around 100 times below than ECG signals. As a result, many types of noise can easily influence them. Because EEG signals are so important in detecting brain illnesses, it is critical to preprocess them for accurate assessment and detection. The crown of your head The EEG is a weighted combination of the signals generated by the different small locations beneath the electrodes on the cortical plate. The rhythm of electrical impulses is useful for evaluating a broad range of brain diseases. Hypertension, Alzheimer, and brain damage are all possibilities. We can compare and distinguish the brainwaves for different emotions and illnesses linked with the brain by studying the EEG signal. Multiple research studies and methodologies for preprocessing, extraction of features, and evaluation of EEG data have recently been created. The use of EEG in human-computer communication could be a novel and demanding field that has acquired traction in recent years. We present predictive modeling for analyzing the customer's preference of likes and dislikes via EEG signal in our report. The impulses were obtained when clients used the Internet to seek for multiple items. The studies were carried out on a dataset that included a variety of consumer goods.

## 1. Introduction

An electroencephalogram (EEG) may be a test want to evaluate the electrical activity within the brain. The communication of brain cells are known as electrical impulses. EEG had been invented by Hans Berger in 1924. An electroencephalogram is used to detect abnormalities in the electrical functioning of the brain. The measurements given by an EEG help us to analyse and rule out various conditions that are related to brain related disorder. The electroencephalogram is a noninvasive approach of measuring brain impulses across the scalp. Electrical measurements from the base of the brain, or possibly the external part of the top, show that the brain has a constant electrical impulse. The neural activity is usually erratic, and no precise patterns can be found in the EEG. EEG signals have a number of benefits, including the fact that they are noninvasive and may be captured in near-real-time. Alpha (8 to 14 Hz), theta (4 to 8 Hz), beta (14 to 30 Hz), Delta (0.5 to 4 Hz), and gamma (14 to 30 Hz) are the five frequencies of an EEG signal (above 30 Hz). In the time-frequency, statistical parameters such as average, mean, variance, sample variance, deviation, kurtosis, and others are used. The most commonly employed bandwidth characteristics in all domains of Electroencephalogram (e.g., analysis are comparative strengths of specific frequencies).

The contributions are listed as follows: the top of the head the electroencephalogram *G* is the average of the signals created by the several tiny regions of the surface of the brain underlying the electrodes. The arrangement of electrical stimulation is useful for recognizing a wide range of brain disorders. Schizophrenia, Alzheimer, and neurological disorders are examples of possible illnesses. We can compare and discriminate the signals generated by the brain for various mood swings and disorders linked with the brain by studying the EEG signal.

## 2. Literature Survey

The fundamentals of EEG impulses and their implications are presented in this study [1]. It provides a critical examination of EEG artefacts techniques and their use in everyday life. The authors of [2] used EEG signals to capture the preferences of ten customers while they saw the goods on a desktop computer. Participants were offered a pair of the identical products in their second experiment, and EEG impulses were acquired. They discovered a rise in the N200 element in the midfrontal electrode, as well as a link between theta band strength and favored items [3]. In Decision modeling and the cerebral: a study on the electroencephalogram of recommendations, the authors looked at the brainwaves of 18 people while they were making private preferences and criticisms for a set of items. An eye tracker was used to capture the user's decision from a sequence of three images displayed on the computer monitor, while EEG pulses were captured simultaneously. The authors examined the alterations in the key bandwidths using PCA and FFT. A significant change in periodic activity with bands was obtained in the temporal, regional, and cerebral areas when the users were signalling their decision by assessing the correlation between the clients choice and various EEG frequencies. [4] Brain data was utilized to develop a premarket prediction method for items. While examining various shoes on a computer monitor, the researchers acquired EEG data from 40 people. After each presentation, the respondents were informed whether they desired to purchase the shoes or otherwise, and they were also provided a survey to complete out utilising a rating range of 1 to 5. Whenever 30 pairs of sneakers were split into two classes, the scientists observed that identification of items using EEG impulses was superior than identification based on ratings, which were estimated as 80 percent and 60 percent, respectively [5]. The researchers investigated the effects of perception on brain systems by studying differences in EEG vibrations, whereas projecting two shades on a desktop computer for one second to nineteen participants in Impact of perceptual colour preference on attention-related temporal oscillations. They discovered a rise in *θ* magnitude when the user's chosen colour was offered or selected. The scientists used EEG signals and an eye tracker device to offer different option sets to 18 participants in order to explore the varied brain processes during decision making. The frequency of responses was recorded using an eye tracker. The most relevant brain areas engaged with the selection task were revealed by a mutual association involving the recovered sensing attributes and the appropriate class value of choice. They identified high theta, alpha, and beta band wave convergence between symmetrical temporal and posterior brain regions, as well as high *θ*, beta, and alpha band frequencies [6].

The researchers suggest the training evaluation method based on EEG signals, which involves establishing a link between neural activity and software readability. They explored if the software could be trained by those who have a lot of alpha waves. ICA was used to segregate multidimensional information into additive constituent parts after capturing electro-cortical stimulation from 120 channels. The scientists discovered patterns in the sensorimotor zone that were engaged in movement execution and association [7]. The researchers recommend utilising the S-Golay filter to eliminate eye blink distortions from EEG signals in real time. They discovered the proper blink signal prediction with a high relationship to the actual blinking signal. The blinking effect was then removed from the EEG signals using an adaptive noise reduction method. Based on the results of the above-given analysis, we use the Savitsky–Golay filtering procedure for filtering, the WDT for retrieval of attributes, and the ANN algorithm for electroencephalogram pulse classification in our work, as these methods are more beneficial and efficient for EEG signal analysis.

EEG has a number of drawbacks. The most significant flaw is its lack of spatial resolution. Its EEG is particularly sensitive to postsynaptic productive things in the cortex's basal layer, on the crests of gyri directly abutting the skull, and radially toward the skull.

## 3. Proposed Method

**Figure 1:**
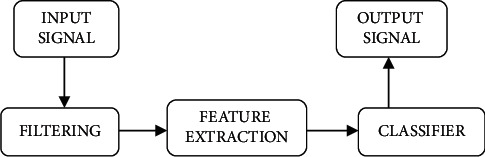
Block Diagram for Analysis of EEG signal using AI IV.

Advertising and publicity of various commercial products via advertising campaigns is a well-documented strategy for increasing sales and public awareness. A generating unit's profit increases as a result of this. Product reproduction is frequently based on a variety of factors, such as market requirements, customer feedback, and rankings, among others. However, “neuro-marketing” refers to the use of subconscious processes to determine consumer interests and forecast conduct in order to successfully use a product. The marketing industry invests a significant amount of money in promoting product usability and success. This is frequently required throughout the pretesting of many potential marketing campaigns prior to launch, as well as during the campaign's postlaunch in-market study. There are a variety of traditional methods for pretesting commercials in Figure 1. These tactics include self-reported tactics such as like, recall, or marketing desire, as well as neuromarketing. The term “neuro-marketing” is made up of the words “neuro” and “marketing.” This will be considered a fusion of several fields, including “neuroimaging” as well as “branding.” Using neuroscience, it is possible to optimize the promotion of existing products. It gives information that can be utilized to improve planning before they are released into the market. As a result, neuroscience gives knowledge regarding product preferences. Using traditional approaches like surveys, views, or conversation, is frequently impossible. As a result, neuromarketing can assist product firms in reducing their advertising spending.

## 4. Materials and Method

### 4.1. Electrodes

The electrodes, which are little metal discs, are positioned in certain locations. The recordist determines these locations by measuring the top using the Global 10/20 System. This is accomplished by calculating the distance between two fixed points on the surface. The probes are then implanted at sites that are ten percent of the width between them and twenty percent of the width between them. A sign, as well as a spectrum of symbols, identifies each electrode site. The letter represents the area of the brain below the electrode 10/20 Scheme of electrode implantation.

1. F-indicates the frontal lobe part of the brain
2. T-represents the temporal lobe part of the brain
3. C-represents the central lobe part of the brain
4. P-indicates the parietal lobe part of the brain
5. O-represents the occipital lobe part of the brain

### 4.2. EEG Activity

The EEG's pulse is typically broken down into the following five unique frequency bands:

1. Beta activity > 13 Hz
2. Alpha activity 8 Hz–13 Hz
3. Theta activity 4 Hz–7 Hz
4. Delta activity < 4 Hz
5. Gamma activity > 30 Hz Regardless of whether the eyelids are opened or shut, beta movement is widespread. This is more typical in streams collected from the center or front of the top. The quantity of beta movement in the waveform is increased by some drugs.

Alpha stimulation is a common activity in aware adults. It is mostly visible in the channels captured from the top's back. It has a frequency of 40 to 100 volts and is generally symmetrical. When the eyes are shut, it is just visible, and when they open, it vanishes or shrinks in amplitude.

**Figure 2:**
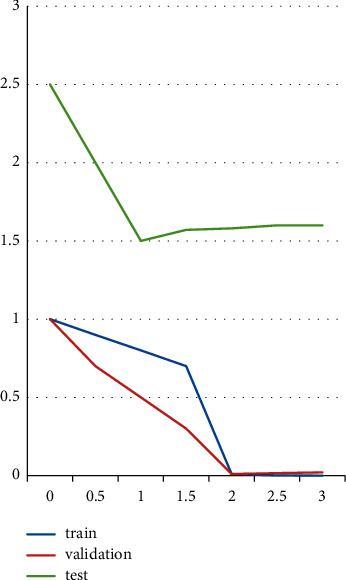
Performance graph.

Depending on the patient's age and health, theta activity is often classified as both a normal and pathological activity in Figure 2. It is very normal for an adult to be sleepy. When noticed in a patient who is aware and conscious; however, it can indicate brain malfunction. Theta activity may also be the most active in channels obtained from the back and center of the top in younger patients.

If an elderly individual is sleeping in a modest to the deep state, delta activity is normal. If it is noticed at a different period, it could indicate brain abnormalities. Depending on the fundamental brain condition, the anomalous activity could be noticed across the board or on specific channels.

There are a range of additional waveforms that are more relevant to specific situations. For example, spike and wave activity Peak and waves activity, for example, suggests a seizure condition. For example, regardless of the individual is not convulsing, signals a seizing diagnosis and should be apparent on the EEG. If spikes or sharp waves are observed, other epileptic diseases could be suspected.

When a patient has a serious liver or kidney illness that affects brain activity, triphasic waves might occur. These are just a few of the most common patterns detected in EEG measurements. It is feasible to mix and match any of the aforementioned patterns, which could contribute to making it challenging to decipher the data. Improper behavior is not usually associated with a single illness, and it could point to a few alternative diagnoses. GBA is a sampling rate in the EEG that ranges from thirty to two hundred Hz and is widely scattered over the skull. GBA is involved in a number of cognitive processes, including vision, focus, memory, consciousness, neuroplasticity, and control.

### 4.3. Savitzsky–Golay Filter

The Savitsky–Golay filter is a least square flattening filter and a computerized nonlinear filter. SG flattening filters are frequently employed in biological signal enhancement and are chosen because they can recover the original signal throughout the noise reduction procedure. Savitzky and Golay created SG filters in 1964, which not only perform preprocessing but also signal smoothing. This filter is based on the linear least square method and is applied to input without modifying the source data.

#### 4.3.1. Equation

The filter's concept is to produce 2N + 1 points that are equally distant from the sequence's center at *N* = 0 for expressing any quadratic with a value. From a subcategory of these equal distance datasets, the equation of the least square polynomial is calculated, and then these polynomials are combined, yielding a number of neural coefficients. The result extracted from the SG filter can be identified by utilising the following equation:

(1) $$\begin{array}{c} \text{SGi}=\sum_{j=-\text{d}-1/2}^{j=\text{d}-1/2}AjBi\;+1\frac{\text{d}+1}{2}\leq i\leq n-\frac{\text{d}-1}{2}. \end{array}$$

The amount of convolutional coefficients are indicated by *d*, while the convolutional coefficients are indicated by A and B. Three elements influence the efficiency of SG filters in practise: the incoming signal, the order of the polynomial, and the frame duration of the polynomial. The source signal as well as the messy portion make up the input signal.

### 4.4. Discrete Wavelet Transform

On discrete wavelet transforms, the wavelet reduction technique has a wide range of applications. On the bottom of the multiresolution feature description, it is been defined. Every dimension taken into consideration reflects a single EEG signal width. The raw EEG *x*(*n*) information was fragmented into many resolutions. 2 digital filters, g(*n*) and *h*(*n*), and 2 down samples are included in each stage. In nature, the discontinuous mother g(*n*) wavelet could be a high pass, whereas its perception *h*(*n*) could be a low pass. Each stage's result offers a signal information and an estimate of the signal, with the most latest being an input for the next phase. The major subband of the EEG data specifies the number of phases to which the wavelet standard is applied.

**Figure 3:**
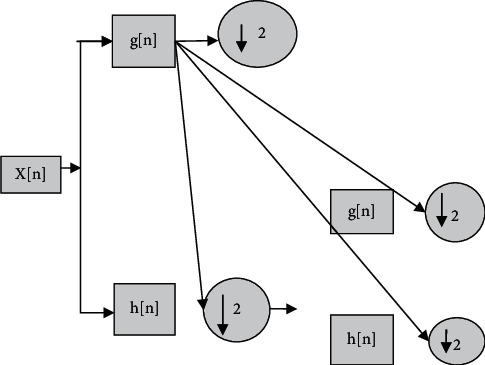
Discrete wavelet transform.

The following diagram depicts the link between WTs and the low pass filter in Figure 3.

(2) $$\begin{array}{c} H\left(z\right)\;H\left(z^{\left(-1\right)}\right)+\;H\left(-z\right)H-\left(z^{\left(-1\right)}\right)=1. \end{array}$$

The h-z-transform of the filter is indicated by *H* (*z*). The z-transform complementary for a high-pass filter is written as follows:

(3) $$\begin{array}{c} G\left(z\right)=zH\left({-z}^{\left(-1\right)}\right). \end{array}$$

There are various perks to the standard convolution-based design of the Wavelet transform that exceed the significant processing and storage demands, such as appropriately characterising the features of the waveform element across a specific bandwidth range and specialised time bandwidth qualities. A multiscale illustration of the sampled value on a limited number of dimensions ranging from best to worst.

#### 4.4.1. Mallat Algorithm

In order to address the wavelets transform in terms of the time varying problem, the Mallat algorithm is used. The decomposition produced includes two outcomes as an approximation (*A*) a low frequency component coefficient and the Detailed (*D*) a high-frequency component coefficient.

### 4.5. Ann Classifier

In untrained people, an ANN was used to evaluate and identify electroencephalographic rhythms related to motor imagery. The complexity of the dataset is reduced by using a classifier for the analysis. Using continuous EEG captured by a collection of thirty-one electrodes structured in accordance with the extended global 10–10 approach, we select an optimum variety of Artificial Neural Network that achieves 80% efficiency for single trial categorization.

Then, by limiting the range of EEG waves to eight in the lobe, we may reach a high level of recognition rate (up to 73 15 percent). Furthermore, we look at the signals time pattern and find that motor-related aspects associated with the left and right foot motor systems are higher prevalent in mu (8–13 Hz) and delta (1–5 Hz) neural impulses than in high-frequency beta (15–30 Hz) neural impulses. Based on the given results, we propose that the EEG data be pretreated including an LP filter by changing limit values to improve the ANN. We show that suppression of high-frequency band elements improves recognition effectiveness greatly (up to 90% correctness using only 8 electrodes). The acquired outcomes are extremely significant in the context of the central nervous system since they contain feed forward neural network responses.

**Figure 4:**
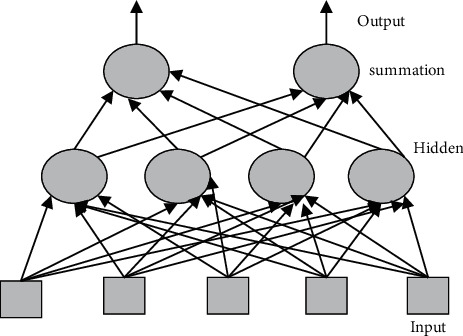
Feed forward neural network.

The quintessential deep neural networks are Deep feedforward networks, also referred to as feed-forward networks or MLPs, which are a type of feedforward network. The basic purpose of this NN is to replicate some parameter *f*^*∗*^. In a classifier, for example, *y* = *f*^*∗*^(*x*) converts a source vector *X* to a group Y by defining a mapping *y* = *f*(*x*) and understanding the value of the parameters that finish in the simplest faster convergence; a feedforward network acquires the value of the specifications that end in the basic function optimization. These frameworks are known as feedforward designs because communication occurs through the function being assessed from *x*, through the additional computations done to define *f*, and ultimately to the result *y*. Because there are insufficient feedback linkages, the model's output is never returned to it. RNNs were feed forward NN with feedback connections that have been adjusted in Figure 4.

## 5. Result and Discussion

The outcomes of the selection prediction calculated over the data obtained to test the proposed system are presented in this section. The input parameters are described in terms of. 11709 clients are given the opportunity to select their favourite and least favourite goods from a list of 14 options on an xls sheet. In our project based on the EEG counter which data have been mentioned in the dataset, and it is been classified as 0–256 EEG counters as a set (i.e., 1–45 customers data are taken) and their respective 14 products filtered and feature extracted signals are analysed.

In our project, we have implemented Matlab coding for the filtering and feature extracting for input data set analysis for all the 45 customers of 14 products. Here, the customer selection is done we have taken customer-5.

The comparison of the input signal from the dataset &the smoothened Savitzky–Golay filter's output. With the help of the Discrete Wavelet transform feature extraction the separation of brainwaves based on the frequency ranges as gamma, beta, alpha, theta, and delta. This product-1 of customer-5 is SHIRT& it is Liked by the customer-5. The amplitude range is from 4100–4200.

The comparison of the input signal from the dataset &the smoothened Savitzky–Golay filter's output. With the help of the Discrete Wavelet transform feature extraction the separation of brainwaves based on the frequency ranges as gamma, beta, alpha, theta, and delta. This product-2 of customer-5 is SHOE and it is liked by the customer-5. The amplitude range is from 4100–4200.

The comparison of the input signal from the dataset &the smoothened Savitzky–Golay filter's output. With the help of the Discrete Wavelet transform feature extraction the separation of brainwaves based on the frequency ranges as gamma, beta, alpha, theta, and delta. This product-3 of customer-5 is SCHOOL BAG& it is liked by the customer-5. The amplitude range is from 4160–4180.

The comparison of the input signal from the dataset &the smoothened Savitzky–Golay filter's output. With the help of the Discrete Wavelet transform feature extraction the separation of brainwaves based on the frequency ranges as gamma, beta, alpha, theta, and delta. This product-4 of customer-5 is TIE& it is Disliked by the customer-5. The amplitude range is from 4150–4174.

The comparison of the input signal from the dataset &the smoothened Savitzky–Golay filter's output. With the help of the Discrete Wavelet transform feature extraction the separation of brainwaves based on the frequency ranges as gamma, beta, alpha, theta, and delta. This product-5 of customer-5 is MUFLER and it is Disliked by the customer 5. The amplitude range is from 4150–4170.

The comparison of the input signal from the dataset &the smoothened Savitzky–Golay filter's output. With the help of the Discrete Wavelet transform feature extraction the separation of brainwaves based on the frequency ranges as gamma, beta, alpha, theta, and delta. This product-6 of customer-5 is BELT& it is Liked by the customer-5. The amplitude range is from 4100–4200.

The comparison of the input signal from the dataset &the smoothened Savitzky–Golay filter's output. With the help of the Discrete Wavelet transform feature extraction the separation of brain waves based on the frequency ranges as gamma, beta, alpha, theta, and delta. This product-1 of customer-5 is BRACELET& it is Liked by the customer-5. The amplitude range is from 4160–4190.

The comparison of the input signal from the dataset &the smoothened Savitzky–Golay filter's output. With the help of the Discrete Wavelet transform feature extraction the separation of brain waves based on the frequency ranges as gamma, beta, alpha, theta, and delta. This product-8 of customer-5 is GLOVE& it is Liked by the customer-5. The amplitude range is from 4160–4200.

The comparison of the input signal from the dataset &the smoothened Savitzky–Golay filter's output. With the help of the Discrete Wavelet transform feature extraction the separation of brain waves based on the frequency ranges as gamma, beta, alpha, theta, and delta. This product-9 of customer-5 is SUNGLASS and it is liked by the customer-5. The amplitude range is from 4150–4200.

The comparison of the input signal from the dataset &the smoothened Savitzky–Golay filter's output. With the help of the Discrete Wavelet transform feature extraction the separation of brain waves based on the frequency ranges as gamma, beta, alpha, theta, and delta. This product-10 of customer-5 is SWEATER and it is Disliked by the customer-5. The amplitude range is from 4160–4172.

The comparison of the input signal from the dataset &the smoothened Savitzky–Golay filter's output. With the help of the Discrete Wavelet transform feature extraction the separation of brain waves based on the frequency ranges as gamma, beta, alpha, theta, and delta. This product-11 of customer-5 is SOCKS& it is Liked by the customer-5. The amplitude range is from 4180–4200.

The comparison of the input signal from the dataset &the smoothened Savitzky–Golay filter's output. With the help of the Discrete Wavelet transform feature extraction the separation of brain waves based on the frequency ranges as gamma, beta, alpha, theta, and delta. This product-12 of customer-5 is WALL CLOCK& it is Disliked by the customer-5. The amplitude range is from 4230–4240.

The comparison of the input signal from the dataset &the smoothened Savitzky–Golay filter's output. With the help of the Discrete Wavelet transform feature extraction the separation of brain waves based on the frequency ranges as gamma, beta, alpha, theta, and delta. This product-13 of customer-5 is PEN and it is Liked by the customer-5. The amplitude range is from 4100–4200.

The comparison of the input signal from the dataset &the smoothened Savitzky–Golay filter's output. With the help of the Discrete Wavelet transform feature extraction the separation of brain waves based on the frequency ranges as gamma, beta, alpha, theta, and delta. This product-1 of customer-5 is WRIST WATCH and it is Liked by the customer-5. The amplitude range is from 4150–4200.

Based on the validation process, Regression analysis and Iteration in the Neural network the likes of the customers are categorized. The products 1, 2, 3, 5, 6, 7, 8, 9, 11, 13, and 14 are liked by the customer-5.

The variables are utilized to assess the proposed technique's performance of the classifier.

45 participants from 14 different items are taken and analysed as part of the validation procedure. This will be the case for all of the dataset's other subsets. The ANN results have been completed, and the output is as follows.

Feed Forward Neural Network Design is used to train neural networks, which uses a back propagation technique. The progress details are Epoch-1000; Performance-1.01e^ (−06); Gradient-1.00e^ (07); Validation Checks – 1.

The learning, verification, testing, best value, and objectives are plotted on the effectiveness graph. At epoch-2, the greatest validation efficiency is 0.011752 in Figure 2.

**Figure 5:**
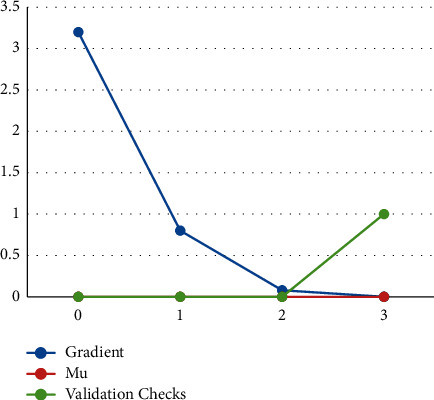
Graph of train status.

In the train state graph, the parameters at epoch-3 are Gradient-0.0098882; Mu-1e-06; Validation Checks-1 in Figure 5.

**Figure 6:**
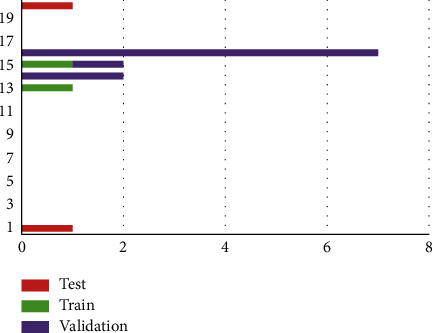
Error histogram.

From Figure 6, the Error Histogram Graph, the training error is at the range (−0.1177 to 0.006746) and instances are from 2–7, the validation error is at the range (−0.1799 to -0.05545) and instances are from 1–2, the testing error is from (−0.9263 to 0.2556) at 1 instance. The Zero error is at 0.006746. The regression plot the values are estimated as the Training: *R* = 0.99559; The Validation: *R* = 1; The Testing: *R* = 1; Overall Range: *R* = 0.84287, where *R* = Regression value.

## 6. Conclusion

In this research, we used EEG signals to forecast a user's purchase decision preference using neuroscience. While seeing products, the brain activity of 45 participants (25 men and 20 women) was monitored. After that, the waveforms were flattened and analysed with a Classification algorithm. The outcome demonstrates the feasibility of the suggested methodology and offers a supplement to traditional methods for estimating product performance in the market. By expanding existing models, the methodology might be used to build market strategy, conduct research, and forecast market success. Furthermore, a neutral option for items could be utilized to offer users with more options. The monitoring of a user's eye mobility while seeing merchandise could be another factor in determining favorite selections. To improve the prediction outcomes, more robust characteristics and classifier configurations should be investigated [8–13].

## Figures and Tables

**Algorithm 1:**
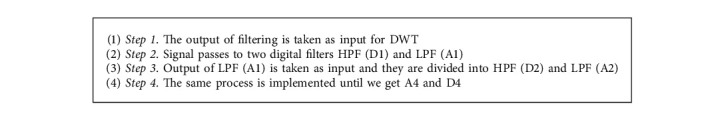
Steps involved in Mallat's algorithm.

## Data Availability

The data used to support the study are included in the paper.
